# Supplementary material for: Association of Vasopressors Dose Trajectories with Enteral Nutrition Tolerance in Patients with Shock: A Prospective Observational Study
Source: Nutrients. 2022 Dec 19;14(24):5393. doi: 10.3390/nu14245393 (PMC9782535; doi:10.3390/nu14245393)
Supplement: Supplementary file 1 [file nutrients-14-05393-s001.zip › nutrients-2080498-supplementary.pdf]

Supplementary Materials

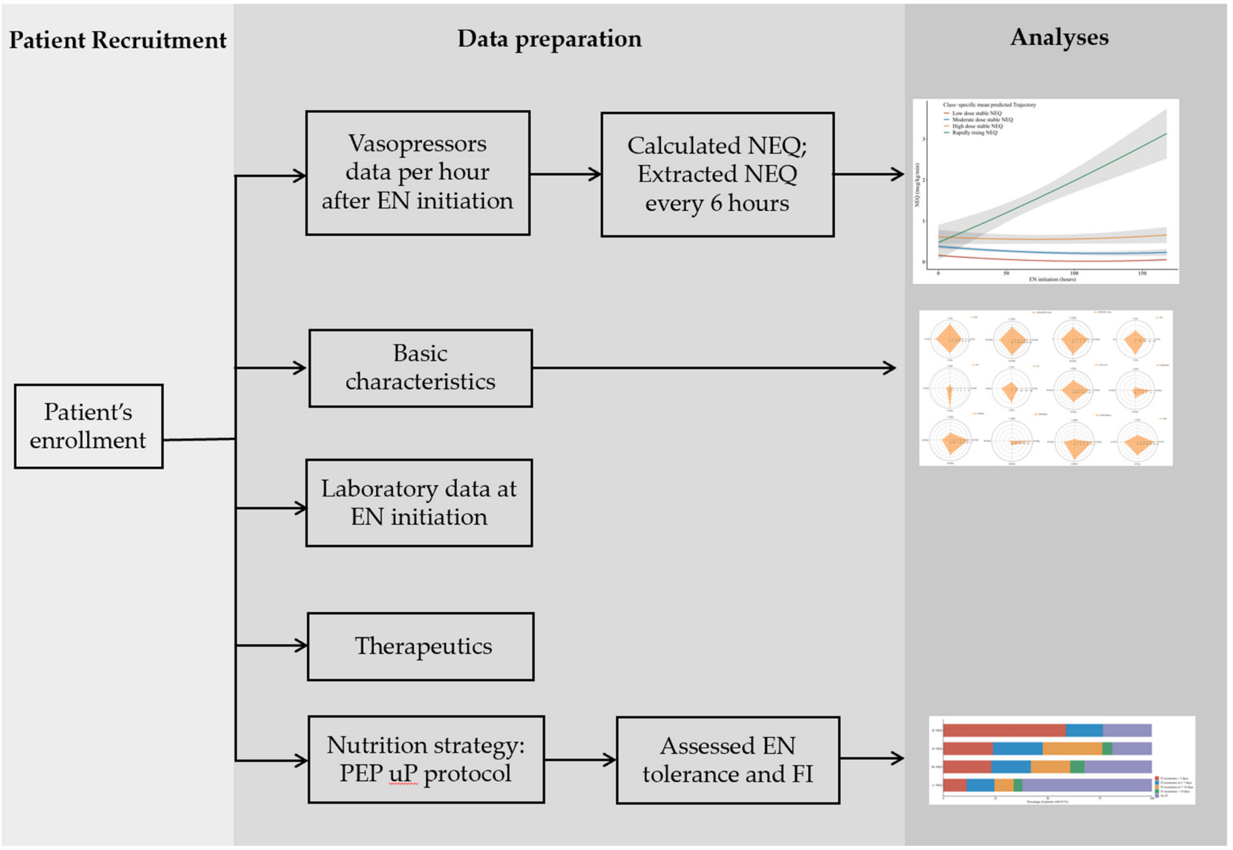

Figure S1. Simplified workflow of study. NEQ: norepinephrine equivalent dose; EN: enteral nutrition; FI: feeding intolerance.

Table S1. Growth Mixed Model results of NEQ model fitting progress.

No. Latent class	Polynomial degree	Log-Lik	BIC	AIC	Entropy	Relative entropy	% Participants per class	Mean posterior probabilities	% Posterior probabilities > 70%
1	Linear	-977.4762	1987.035	1966.95	NA	NA	100	NA	NA
1	Quadratic	-944.5013	1926.432	1903	NA	NA	100	NA	NA
1	Cubic	-2046.881	4136.539	4109.76	NA	NA	100	NA	NA
2	Linear	-746.6801	1546.831	1513.360	21.672	0.851	54.76/45.23	0.946/0.979	94.78/95.79
2	Quadratic	-713.8582	1486.535	1449.717	21.717	0.851	54.76/45.23	0.948/0.975	94.74/94.79
2	Cubic	-2029.302	4122.768	4082.603	144.595	0.007	88.57/11.43	0.540/0.540	0.00/4.17
3	Linear	-652.2548	1379.369	1332.510	34.113	0.852	48.57/9.05/42.38	0.928/0.963/0.948	92.16/94.74/95.51
3	Quadratic	-619.0748	1318.356	1268.150	34.117	0.852	42.38/48.57/9.05	0.950/0.927/0.964	95.51/92.16/94.74
3	Cubic	-2022.968	4131.490	4077.936	228.948	0.008	29.52/65.24/5.24	0.373/0.362/0.445	0.00/0.00/0.00
4	Linear	-639.1103	1374.469	1314.221	43.402	0.851	46.67/8.10/35.24/10.00	0.938/0.991/0.887/0.933	94.90/100.00/87.84/95.24
**4**	**Quadratic**	**-605.8501**	**1313.295**	**1249.700**	**43.356**	**0.851**	**35.24/46.67/10.00/8.10**	**0.888/0.937/0.933/0.991**	**89.19/94.90/95.24/100.00**
4	Cubic	-2025.599	4158.140	4091.198	288.179	0.010	2.86/59.52/32.86/4.76	0.338/0.281/0.284/0.380	0.00/0.00/0.00/0.00
5	Linear	-628.0249	1373.686	1300.050	53.642	0.841	46.19/10.48/8.10/25.71/9.52	0.924/0.795/0.994/0.891/0.903	89.69/81.82/100/88.89/90
5	Quadratic	-594.6466	1312.277	1235.293	53.816	0.841	46.19/10.48/25.71/8.10/9.52	0.923/0.796/0.891/0.993/0.904	87.63/81.82/87.04/100/90
5	Cubic	-2025.492	4179.315	4098.984	333.125	0.014	1.90/28.57/49.05/17.14/3.33	0.299/0.237/0.233/0.246/0.363	0.00/0.00/0.00/0.00/0.00
6	Linear	-628.0249	1395.075	1295.892	116.871	0.689	12.38/15.71/8.10/45.24/10.47/8.10	0.831/0.863/0.822/0.925/0.949/0.996	80.77/84.85/82.35/87.37/95.45/100
6	Quadratic	-588.5445	1321.461	1231.089	56.181	0.851	12.38/45.24/8.10/8.10/15.71/10.47	0.834/0.924/0.822/0.996/0.860/0.950	80.77/87.37/82.35/100/84.85/95.45
6	Cubic	-2027.606	4204.930	4111.211	368.807	0.020	1.43/1.43/59.52/29.05/5.71/2.86	0.262/0.227/0.208/0.208/0.226/0.338	0.00/0.00/0.00/0.00/0.00/0.00

NEQ: norepinephrine equivalent dose; BIC: Bayesian information criterion; AIC: Akaike information criterion; NA: Not acquire.

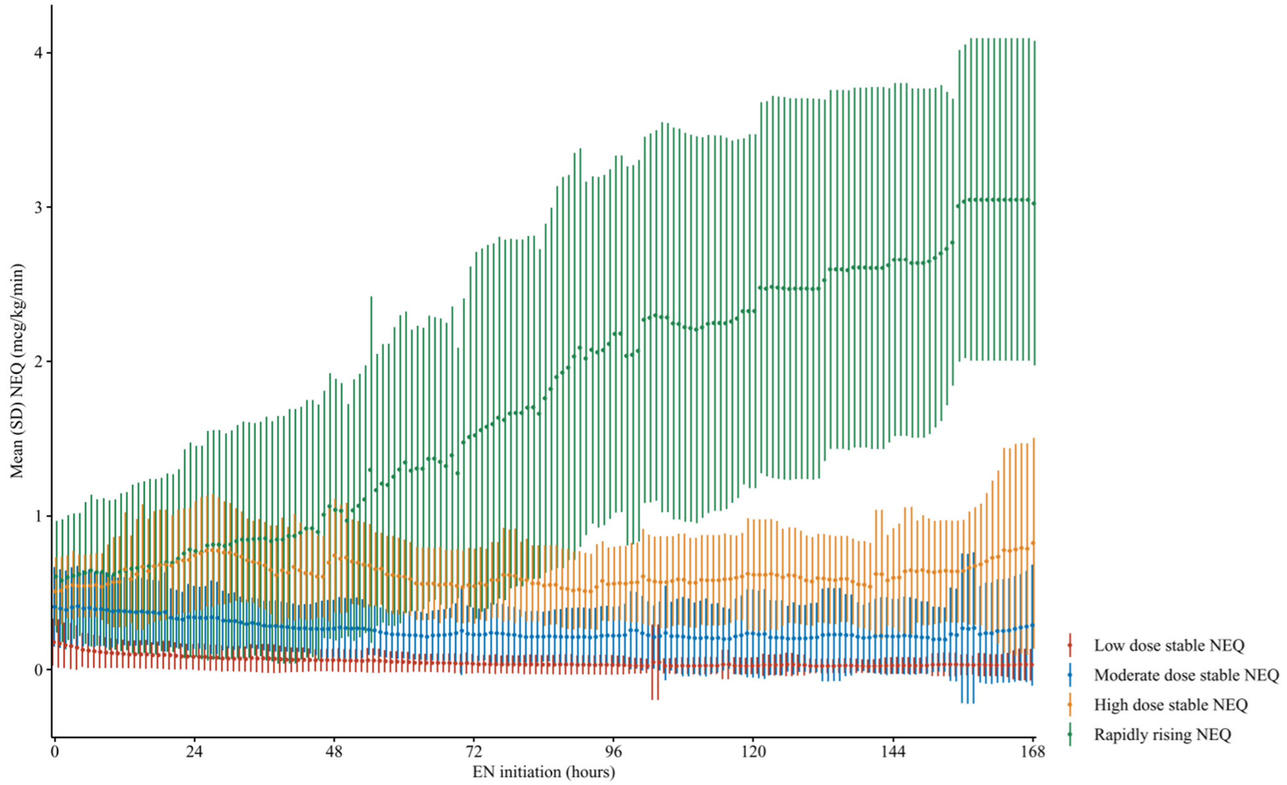

Figure S2. The dynamics of NEQ use in patients with shock across NEQ Trajectory Groups. EN, enteral nutrition; NEQ, norepinephrine equivalent dose; SD, standard deviation

Table S2. Basic characteristics of patients with or without FI in each group.

	L-NEQ				M-NEQ				H-NEQ				R-NEQ			
	Total (n=98)	Non-FI (n=61)	FI (n=37)	P value	Total (n=74)	Non-FI (n=24)	FI (n=50)	P value	Total (n=21)	Non-FI (n=4)	FI (n=17)	P value	Total (n=21)	Non-FI (n=4)	FI (n=13)	P value
**Age (years)**	63 (48-71.3)	66 (51-74)	53 (38.5-66.5)	0.007	64.5 (52-70)	58.5 (49.3-71.5)	66 (52.8-69.3)	0.521	67 (53.5-76.5)	49 (46-70.8)	70 (58.5-76.5)	0.139	62 (45.5-73)	67.5 (48-72)	55 (45.5-73.5)	0.692
**Male Sex—no.(%)**	66 (67.35)	41 (67.21)	25 (67.57)	0.971	47 (63.51)	16 (66.67)	31 (62)	0.696	14 (66.67)	3 (75)	11 (64.71)	>0.999	14 (82.35)	4 (100)	10 (76.92)	0.541
**BMI**	23.9 (21.5-25.8)	23.9 (22.1-26.5)	23 (20.7-25.6)	0.218	22.3 (19.9-24.7)	21.3 (19.5-23.4)	23 (20-25)	0.077	22 (19.5-23.8)	21.5 (19.5-24.6)	22 (19.1-23.8)	0.823	18.7 (17.2-23.5)	21.4 (17.8-25)	18.7 (16.9-22.1)	0.257
**APACHE II score**	20.9 (15-26)	21 (16-26)	19 (14.5-25.5)	0.287	20 (12.8-25.3)	17 (10.5-27)	20 (13.8-25.3)	0.374	25 (15.5-27.5)	12.5 (10.3-21.5)	26 (18-28)	0.025	24 (12-29)	29.5 (16-31)	21 (12-27)	0.189
**NRS2002 score (IQR)**	3 (3-4)	3 (3-4)	3 (3-4)	0.138	3 (3-4.3)	3.5 (3-6)	3 (3-4)	0.146	4 (3-5.5)	3 (3-3.8)	4 (3-6)	0.110	4 (3-6)	6 (3.8-6.8)	4 (3-6)	0.239
**Diagnose—no.(%)**																
**Severe pneumonia**	34 (34.69)	22 (36.07)	12 (32.43)	0.714	38 (51.35)	14 (58.33)	24 (48)	0.405	13 (61.9)	3 (75)	10 (58.82)	0.978	7 (41.18)	1 (25)	6 (46.15)	0.603
**ARDS**	10 (10.2)	6 (9.84)	4 (10.81)	>0.999	4 (5.41)	2 (8.33)	2 (4)	0.824	3 (14.29)	0 (0)	3 (17.65)	0.910	3 (17.65)	1 (25)	2 (15.38)	>0.999
**MODS**	14 (14.29)	7 (11.48)	7 (18.92)	0.307	8 (10.81)	2 (8.33)	6 (12)	0.940	2 (9.52)	0 (0)	2 (11.76)	>0.999	5 (29.41)	0 (0)	5 (38.46)	0.261
**Liver disease**	9 (9.18)	8 (13.11)	1 (2.7)	0.147	3 (4.05)	1 (4.17)	2 (4)	>0.999	2 (9.52)	0 (0)	2 (11.76)	>0.999	3 (17.65)	0 (0)	3 (23.08)	0.541
**Cardiac arrest**	14 (14.29)	8 (13.11)	6 (16.22)	0.671	6 (8.11)	2 (8.33)	4 (8)	>0.999	2 (9.52)	0 (0)	2 (11.76)	>0.999	2 (11.76)	1 (25)	1 (7.69)	0.426
**Cardiovascular disease***	57 (58.16)	35 (57.38)	22 (59.46)	0.839	46 (62.16)	16 (66.67)	30 (60)	0.58	14 (66.67)	3 (75)	11 (64.71)	0.694	13 (76.47)	2 (50)	11 (84.62)	0.219
**Neurological disease†**	42 (42.86)	31 (50.82)	11 (29.73)	0.041	30 (40.54)	9 (37.5)	21 (42)	0.712	10 (47.62)	1 (25)	9 (52.94)	0.652	3 (17.65)	1 (25)	2 (15.38)	>0.999
**Types of shock—no.(%)**																
**Sepsis shock**	80 (81.63)	49 (80.33)	31 (83.78)	0.668	62 (83.78)	23 (95.83)	39 (78)	0.051	19 (90.48)	4 (100)	15 (88.24)	>0.999	17 (100)	4 (100)	13 (100)	>0.999
**Cardiac shock**	10 (10.2)	6 (9.84)	4 (10.81)	>0.999	10 (13.51)	2 (8.33)	8 (16)	0.589	1 (4.76)	0 (0)	1 (5.88)	>0.999	1 (5.88)	0 (0)	1 (7.69)	1
**Hemorrhagic shock**	13 (13.27)	9 (14.75)	4 (10.81)	0.802	10 (13.51)	2 (8.33)	8 (16)	0.589	2 (9.52)	0 (0)	2 (11.76)	>0.999	0 (0)	0 (0)	0 (0)	0.429
**Laboratory data, median (IQR)**																
**White blood cell (x10^{9}/L)**	10 (7.6-13.7)	9.7 (7.3-13.8)	10.5 (8.2-13.8)	0.410	11.5 (8.2-14.3)	10 (6.2-14.5)	11.9 (9.4-14.1)	0.184	13.7 (9.3-16.2)	21 (10.7-27.3)	12.8 (9.3-15.3)	0.107	10.4 (4.5-18.8)	18.8 (12.1-28.1)	8.1 (3.2-14.5)	0.042

**Platelet ($x10^9$/L)**	95.5 (55.5-205.3)	86 (53.5-183.5)	113 (55-238)	0.326	109 (61-171)	74.5 (44.8-165)	122 (71.3-187)	0.050	87 (40-129.5)	45 (7.8-104)	101 (52-137.5)	0.152	72 (22.5-130.5)	185 (86.8-241.3)	36 (18-96.5)	0.024
**Hemoglobin (g/L)**	83.5 (72-98.3)	83 (72-102)	87 (70.5-96.5)	0.781	89 (78.8-100.3)	83 (78.3-96.5)	90.5 (79.5-101.8)	0.355	91 (76.5-103.5)	74 (66.5-102.5)	91 (82-103.5)	0.244	87 (74.5-118)	108.5 (82.5-123.3)	84 (71.5-107.5)	0.308
**Albumin (g/L)**	32.3 (29.3-36)	31.6 (29.1-34.7)	34.2 (30.2-37.9)	0.012	31.8 (28.6-34.8)	32.7 (28.7-36)	31.1 (28.6-34.1)	0.453	30.8 (28.8-37.3)	28.7 (21.6-37.1)	32.2 (29.3-37.3)	0.282	34.4 (32.7-37)	36.7 (34.1-38.4)	34.2 (31.7-35.8)	0.234
**Total bilirubin (umol/L)**	16.9 (10.3-32.1)	15 (10-27)	22.7 (10.6-39.9)	0.132	17.9 (8.6-33.7)	18.8 (9.5-34.2)	16.3 (7.6-33.7)	0.738	15.9 (12.3-21.2)	19 (12.3-22.1)	15.8 (12.3-24.8)	0.591	27.6 (15.8-41.2)	14.1 (11.1-24.7)	37.2 (16-48.2)	0.070
**Serum creatinine (umol/L)**	93 (62.8-164.5)	89 (61-166.5)	94 (67-162.5)	0.778	81.5 (57.8-150.8)	81 (50.5-166.3)	82.5 (58.8-144.8)	0.899	100 (72-142.5)	104 (74.5-138.8)	100 (68-159)	0.893	90 (63.5-124.5)	74 (65.3-163.8)	98 (59.5-124.5)	0.821
**Glucose (mmol/L)**	8.4 (6.5-11.4)	8.2 (6.4-11.4)	8.5 (6.6-11.4)	0.517	9.3 (6.7-13.5)	10.8 (7.1-13.8)	9.2 (6.4-13.3)	0.365	10.5 (8.7-12.6)	9.8 (8.8-10.7)	11 (8.6-13.1)	0.420	8.9 (7-11.8)	8.4 (4.7-11.9)	8.9 (8.1-12.1)	0.497
**C-reactive protein (mg/L)**	93 (56.4-136.3)	88.7 (34.4-131.5)	102 (67.1-203.5)	0.088	122.5 (69.1-172.3)	135 (71.4-169)	111.5 (67.2-175.3)	0.742	162 (123-236.5)	193.5 (129-240.8)	162 (116-225.5)	0.622	107 (39.3-178)	148 (99.1-180.5)	78.8 (23-171.5)	0.308
**Procalcitonin (ug/L)**	1.6 (0.4-6.9)	1.3 (0.4-5.1)	2.9 (0.4-11.6)	0.291	1.8 (0.7-7.2)	1.7 (0.8-6.6)	2.2 (0.5-8.3)	0.751	10.3 (0.8-21.7)	8.2 (0.9-39.1)	10.3 (0.8-21.7)	0.858	1.3 (0.7-8.2)	1.1 (0.5-9)	2.8 (0.7-17.5)	0.651
**Interleukin-6 (ug/L)**	64.8 (26.5-151.5)	52.4 (26.5-133.5)	74.3 (29.3-213.2)	0.397	101 (38.6-281.6)	138.6 (42.5-334.5)	101 (32.2-243.5)	0.288	140 (43.9-523.7)	409 (97.2-991.3)	137 (35.7-354.7)	0.244	64.9 (37-168.7)	78 (48.5-835)	64.3 (32.5-168.7)	0.428
**Lactic acid (mmol/L)**	1.5 (1.2-1.8)	1.5 (1.2-1.8)	1.6 (1.2-1.9)	0.749	1.8 (1.4-2.3)	2 (1.5-2.4)	1.8 (1.4-2.2)	0.325	1.8 (1.4-2.5)	2 (1.7-2.8)	1.8 (1.4-2.5)	0.419	2.4 (1.4-3.6)	2.5 (1.7-3.1)	2.4 (1.3-4.5)	0.955
**Arterial partial pressure of oxygen (mmHg)**	104.4 (78.9-122.6)	104 (83.9-129.1)	104.7 (71.8-120.1)	0.383	98.3 (70.7-126.1)	89 (51.7-117.5)	99.7 (75.1-130.2)	0.321	93.5 (62.8-132.8)	83.6 (36.1-149.5)	93.5 (71.3-132.8)	0.591	77.9 (69.2-124.8)	85.5 (69.4-137.6)	77.9 (63.6-124.8)	0.910
**Arterial oxygen saturation (%)**	98.8 (96.5-99.6)	98.9 (97.1-99.6)	98.7 (94.5-99.7)	0.468	98.6 (96.2-99.3)	98.2 (86.8-99.6)	98.8 (96.7-99.3)	0.499	98.5 (91-99.3)	89.4 (76-99.7)	98.5 (92.8-99.2)	0.929	96.8 (93.8-99.3)	97.6 (93.4-99.5)	96.8 (89-99.3)	0.865
**Sedation and analgesia**																
**Midazolam (mg/kg/day)**	0.7±1	0.7±1	0.5±1	0.382	0.7±1.1	0.8±1.3	0.7±1	0.817	1.9±2.6	3.7±5.5	1.5±1.5	0.488	3.8±9.1	11.2±18.4	1.5±1	0.367
**Propofol (mg/kg/day)**	5.5±6.7	6.1±7.6	4.3±4.7	0.151	4.3±5.6	2.7±3.9	5.1±6.2	0.086	5.4±5.5	1.7±3.4	6.3±5.6	0.139	8±8	14±6.7	6.1±7.6	0.084
**Dexmedetomidine (ug/kg/day)**	1.9±2.1	1.8±2.2	2.2±2	0.384	3.1±6.7	2.6±2.6	3.3±7.9	0.663	1.4±2.4	2.8±4.1	1.1±1.9	0.222	2.9±3.6	4.2±5.8	2.4±2.9	0.404
**Remifentanil (ug/kg/day)**	33.8±62.6	41.8±72.1	20.7±40	0.065	40.4±56.8	39.4±69.3	40.8±50.5	0.920	44.1±70.9	24.5±49.1	48.7±75.5	0.553	39.3±58.2	74.8±82.3	28.4±47.7	0.170
**Sufentanyl (ug/kg/day)**	1±1.7	0.9±1.7	1.2±1.9	0.330	1.2±1.9	1±1.9	1.3±1.9	0.512	2.1±2.2	1.5±1.3	2.3±2.3	0.501	2.6±2.9	3±4.4	2.5±2.5	0.747

**Treatment—no.(%)**																
**Norepinephrine at EN initiation (ug/kg/min)**	0.2 (0.1-0.3)	0.1 (0.1-0.2)	0.2 (0.1-0.3)	0.081	0.4 (0.2-0.6)	0.4 (0.1-0.6)	0.4 (0.2-0.6)	0.940	0.4 (0.3-0.6)	0.4 (0.4-0.5)	0.5 (0.3-0.7)	0.720	0.5 (0.4-0.7)	0.4 (0.2-0.5)	0.5 (0.4-1)	0.061
**NEQ at EN initiation (ug/kg/min)**	0.2 (0.1-0.2)	0.1 (0.1-0.2)	0.2 (0.1-0.3)	0.175	0.4 (0.2-0.6)	0.4 (0.1-0.6)	0.4 (0.2-0.6)	0.940	0.4 (0.3-0.6)	0.4 (0.4-0.5)	0.5 (0.3-0.7)	0.720	0.5 (0.4-0.7)	0.4 (0.2-0.5)	0.5 (0.4-1.1)	0.061
**Prokinetics**	53 (54.08)	28 (45.9)	25 (67.57)	0.037	28 (37.84)	5 (20.83)	23 (46)	0.037	10 (47.62)	0 (0)	10 (58.82)	0.090	3 (17.65)	1 (25)	2 (15.38)	>0.999
**CRRT**	30 (30.93)	12 (20)	18 (48.65)	0.003	29 (39.19)	9 (37.5)	20 (40)	>0.999	9 (42.86)	1 (25)	8 (47.06)	0.603	8 (47.06)	1 (25)	7 (53.85)	0.576
**ECMO**	9 (9.28)	5 (8.33)	4 (10.81)	0.683	2 (2.7)	1 (4.17)	1 (2)	>0.999	0 (0)	0 (0)	0 (0)		2 (11.76)	0 (0)	2 (15.38)	>0.999
**Inotropic drugs**	22 (22.4)	14 (23)	8 (21.6)	0.878	10 (41.7)	26 (52)	36 (48.6)	0.405	16 (76.2)	2 (50)	14 (82.4)	0.228	11 (64.7)	1 (9.1)	10 (76.9)	0.099
**Dobutamine (ug/kg/day)**	0±0	0±0	0±0.1	0.324	0.1±0.9	0±0	0.2±1.1	0.400	0.2±0.7	0±0.1	0.3±0.8	0.896	0.6±2.3	0±0	0.7±2.7	0.596
**Cumulative fluid balance at first week (L)**	0.8±4.3	0.8±4.8	0.9±3.6	0.947	2.5 (0.1-5.1)	2.4 (-0.9-4.6)	2.5 (0.2-5.7)	0.496	5.4±8.9	0.9±4.1	6.4±9.5	0.282	5.4±3.9	6.4±1.4	5.1±4.4	0.571

All P values are 2-tailed. Differences was analyzed between patients with and without FI in each trajectory group. APACHE II, Acute Physiology and Chronic Health Evaluation II; ARDS, acute respiratory distress syndrome; BMI, body mass index; CRRT, continuous renal replacement therapy; ECMO, extracorporeal membrane oxygenation; FI, feeding intolerance; IQR, interquartile range; MODS, multiple organ dysfunction syndrome; NRS2002, Nutritional Risk Screening 2002; NEQ, norepinephrine equivalent dose; SD, standard deviation; L-NEQ, low dose stable NEQ; M-NEQ, moderate dose stable NEQ; H-NEQ, high dose stable NEQ; R-NEQ, rapidly rising NEQ

*Cardiovascular diseases are coronary heart disease, hypertension, acute myocardial infarction, infectious endocarditis, arrhythmia, cardiomyopathy, cardiac insufficiency, cardiac failure, and pericardial effusion.

†Neurological diseases are Parkinson's disease, hypoxic-ischemic encephalopathy, epilepsy, cerebral contusion, subarachnoid hemorrhage, intracranial infection, cerebral infarction, brain atrophy, intracranial hematoma, and stroke.

Table S3. Clinical outcomes in patients with or without FI in each trajectory groups.

	**Low dose stable NEQ**				**Moderate dose stable NEQ**				**High dose stable NEQ**				**Rapidly rising NEQ**			
	Total (n=98)	Non-FI (n=61)	FI (n=37)	P value	Total (n=74)	Non-FI (n=24)	FI (n=50)	P value	Total (n=21)	Non-FI (n=4)	FI (n=17)	P value	Total (n=21)	Non-FI (n=4)	FI (n=13)	P value
EN starting time from ICU admission (hours), median (IQR)	25.1 (17.8-46.4)	24.8 (17.7-44.3)	25.8 (18.2-88)	0.174	34.8 (17.1-60.1)	35.3 (15.8-63.6)	33.8 (17.9-59.7)	0.926	35.6 (18.7-72.3)	20.8 (9.7-61.7)	36.7 (19.5-77.3)	0.771	36.7 (20.2-71.8)	22.7 (16.7-33.8)	45 (22.2-96.3)	0.113
The EN intake at first week after EN initiation, median (IQR)																
Energy (kcal/kg)	8 (4.8-11.9)	8 (4.2-12.7)	8 (5.5-10.6)	0.711	9.9 (6.8-14.2)	11.2 (8.4-17.1)	8.9 (5.9-14.4)	0.086	7.7 (4.1-10.8)	10.2 (7.9-13.5)	6.9 (2.6-10.1)	0.128	5.5 (3.4-11)	9 (5.3-12.3)	4.4 (2.8-8.5)	0.174
Protein (g/kg)	0.3 (0.2-0.5)	0.3 (0.2-0.5)	0.3 (0.2-0.4)	0.401	0.4 (0.3-0.6)	0.5 (0.3-0.7)	0.3 (0.2-0.6)	0.071	0.3 (0.2-0.4)	0.4 (0.3-0.6)	0.3 (0.1-0.4)	0.210	0.2 (0.1-0.5)	0.4 (0.2-0.5)	0.2 (0.1-0.3)	0.174
Fat (g/kg)	0.2 (0.1-0.4)	0.2 (0.1-0.4)	0.2 (0-0.4)	0.517	0.3 (0.1-0.4)	0.3 (0.1-0.4)	0.2 (0.1-0.4)	0.301	0.2 (0-0.3)	0.3 (0.2-0.7)	0.1 (0-0.3)	0.128	0.2 (0-0.4)	0.3 (0.3-0.6)	0.1 (0-0.3)	0.042
The total nutrition intake at first week after EN initiation, median (IQR)																
Energy (kcal)	19 (13.9-24.8)	19.1 (12.6-24.7)	18.9 (14.5-25.4)	0.397	20.1 (14.2-25.1)	22.2 (14.6-25.1)	19.3 (12.8-25.8)	0.622	18.6 (10.9-23.9)	18.1 (9.3-30.4)	18.6 (12.3-22.7)	>0.999	16.8 (10.6-23.1)	22.6 (13.3-36.9)	13.2 (8.8-22.2)	0.113
Protein (g)	0.7 (0.5-0.9)	0.7 (0.4-1)	0.7 (0.5-0.9)	0.647	0.6 (0.5-1)	0.7 (0.5-1)	0.6 (0.5-0.9)	0.581	0.6 (0.4-0.9)	0.7 (0.3-1.1)	0.6 (0.4-0.9)	0.858	0.3 (0.2-0.8)	0.7 (0.4-1.5)	0.2 (0.2-0.6)	0.070
Fat (g)	0.7 (0.4-0.9)	0.7 (0.4-1)	0.7 (0.4-0.8)	0.956	0.6 (0.4-0.9)	0.6 (0.4-0.9)	0.6 (0.4-0.9)	0.796	0.7 (0.3-0.9)	0.6 (0.2-1.4)	0.7 (0.3-0.8)	0.788	0.4 (0.2-0.9)	0.9 (0.4-1.7)	0.3 (0.1-0.7)	0.042
MV (day), median (IQR)	12.5 (5.5-23.1)	10.1 (4.7-22)	15.5 (9-33.8)	0.031	15 (9.9-25.3)	11.7 (6.5-16.8)	16.4 (10.5-27.8)	0.031	10.9 (5.4-17.6)	3.7 (3-7.1)	13.1 (9.1-18.2)	0.007	6.6 (5.1-7.7)	5.7 (4.7-7.3)	6.9 (5.2-8.4)	0.213
ICU Length of stay (day), median (IQR)	17.5 (11.8-25.3)	16 (9.3-23.5)	22 (13.8-40)	0.007	17 (12-27.1)	13.6 (12-18.8)	18 (13-29.6)	0.049	12 (7-18.2)	8 (4.3-11.4)	14 (9.8-19.5)	0.044	7.6 (6-9)	7 (6-8.8)	7.6 (6.2-9)	0.690
28-days mortality —no.(%)	15 (15.31)	11 (18.03)	4 (10.81)	0.501	28 (37.84)	9 (37.5)	19 (38)	0.967	13 (61.9)	3 (75)	10 (58.82)	>0.999	17 (100)	4 (100)	13 (100)	

All P values are 2-tailed. Differences was analyzed between patients with and without FI in each trajectory group. EN enteral nutrition; FI feeding intolerance; NEQ, norepinephrine equivalent dose; ICU intensive care unit; IQR, interquartile range; MV mechanical ventilation; L-NEQ, low dose stable NEQ; M-NEQ, moderate dose stable NEQ; H-NEQ, high dose stable NEQ; R-NEQ, rapidly rising NEQ

Table S4. Cox proportional hazard regression analysis of the effect of NEQ trajectory groups on 28-days mortality.

28-days mortality	Model 1 HR (95% CI)	Model 1 *P* Value	Model 2 HR (95% CI)	Model 2 *P* Value	Model 3 HR (95% CI)	Model 3 *P* Value	Model 4 HR (95% CI)	Model 4 *P* Value
Baseline joint groups								
Low dose stable NEQ	1.0 (Ref)		1.0 (Ref)		1.0 (Ref)		1.0 (Ref)	
Moderate dose stable NEQ	2.79 (1.489, 5.228)	0.001	2.614 (1.344, 5.084)	0.005	2.726 (1.399, 5.312)	0.003	2.611 (1.333, 5.113)	0.004
High dose stable NEQ	6.084 (2.887, 12.822)	< 0.001	5.472 (2.416, 12.396)	< 0.001	5.514 (2.46, 12.357)	< 0.001	5.967 (2.625, 13.563)	< 0.001
Rapidly rising NEQ	87.212 (34.882, 218.042)	< 0.001	79.977 (29.734, 215.118)	< 0.001	86.538 (32.283, 231.974)	< 0.001	85.942 (31.063, 237.776)	< 0.001
P for trend	<0.001		<0.001		<0.001		<0.001	
Covariates								
Age (years)			1.022 (1.006, 1.039)	0.009	1.019 (1.002, 1.037)	0.028	1.02 (1.002, 1.038)	0.028
BMI			0.922 (0.859, 0.989)	0.024	0.905 (0.841, 0.974)	0.008	0.901 (0.835, 0.972)	0.007
NEQ at EN initiation (µg/kg/min)			0.69 (0.261, 1.819)	0.452	0.579 (0.225, 1.495)	0.259	0.445 (0.168, 1.18)	0.104
APACHE II score					1.03 (1.001, 1.06)	0.042	1.029 (0.999, 1.06)	0.062
Dobutamine (µg/kg/day)							1.028 (0.869, 1.215)	0.748
Cumulative fluid balance at first week (L)							0.997 (0.958, 1.038)	0.891
Lactic acid (mmol/L)							1.374 (1.17, 1.614)	< 0.001

Model 1 was unadjusted. Model 2 was adjusted for age, BMI and NEQ at EN initiation. Model 3 was adjusted for age, BMI, NEQ at EN initiation, and APACHE II score. Model 4 was adjusted for age, BMI, NEQ at EN initiation, APACHE II score, dobutamine and cumulative fluid balance at first week. APACHE II: acute physiology and chronic health evaluation II, BMI: body mass index, CI: confidence interval, HR: hazard ratio, EN: enteral nutrition, NEQ: norepinephrine equivalent dose.

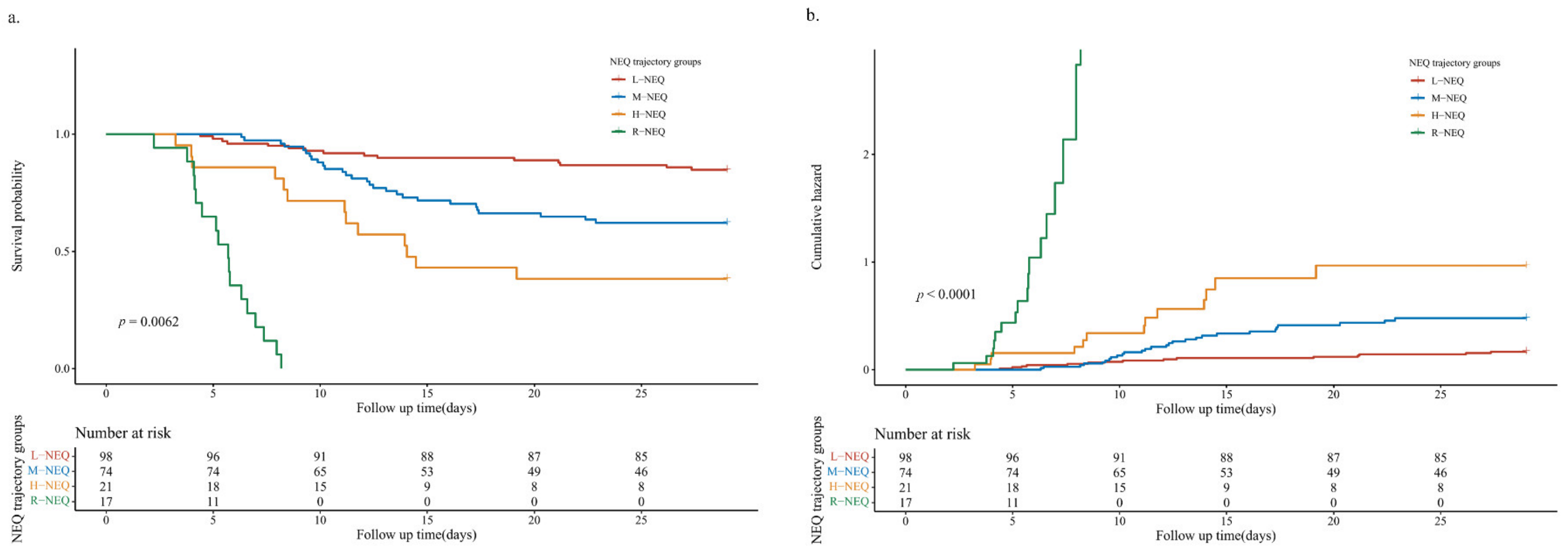

Figure S3. Kaplan-Meier survival curves (**a.**) and cumulative risk (**b.**) of 28-days mortality for NEQ trajectory groups. NEQ, norepinephrine equivalent dose.
